# Improved Coomassie Blue Dye-Based Fast Staining Protocol for Proteins Separated by SDS-PAGE

**DOI:** 10.1371/journal.pone.0081696

**Published:** 2013-11-21

**Authors:** Pavel Májek, Zuzana Riedelová-Reicheltová, Klára Pecánková, Jan E. Dyr

**Affiliations:** Department of Biochemistry, Institute of Hematology and Blood Transfusion, Prague, Czech Republic; Shantou University Medical College, China

## Abstract

The time required to visualize proteins using Coomassie Blue dye has been significantly reduced with the introduction of fast staining protocols based on staining with a Coomassie Blue dye solution at boiling temperatures. However, fast stainings suffer from high gel backgrounds, reducing the signal-to-noise ratio and limiting the number of detectable spots in the case of 2D SDS-PAGE. The aim of this work was to eliminate the high gel background, and thus improve fast staining protocols based on Coomassie Blue dye. We show that merely replacing water with a 4 mM EDTA washing solution at boiling temperatures, results in a transparent gel background within 50 to 60 minutes of destaining. Moreover, when a combination of imidazole-zinc reverse staining and Coomassie Blue-based fast staining is used the sensitivity is improved significantly; nanogram amounts of proteins can be detected using 1D SDS-PAGE, and about 30% to 60% more spots can be detected with 2D SDS-PAGE in plasma, platelet, and rat brain tissue samples. This work represents an optimized fast staining protocol with improved sensitivity, requiring between 60 to 75 minutes to complete protein visualization.

## Introduction

The visualization of proteins separated by SDS-PAGE is one of the crucial steps in gel-based proteomics. There are many protein visualization techniques differing in sensitivity, time required, cost, complication to perform, etc. Coomassie Blue staining (CBS) remains one of the most popular protein stainings due to its simplicity, mass spectrometry compatibility, and low-cost. However, there are other staining protocols more sensitive than CBS [1], [2]; and moreover, using Coomassie Blue dye can be time consuming. The time required to visualize proteins using CBS has been significantly reduced with fast staining protocols utilizing Coomassie Blue dye. Recently, a protocol introduced by Dong *et al*. enabled simple protein visualization, requiring only minutes to finish the staining procedure [3]. Moreover, Dong’s protocol avoids the use of organic solvents, and thus represents an excellent alternative as a simple, timesaving, low-cost, and environmental friendly solution. Nevertheless, the Dong protocol does not seem to be as sensitive as traditional colloidal CBS – a high gel background (reduced signal-to-noise ratio) has been shown to be a limiting factor [4]. Increasing the destaining time from 10 to 12 hours was recommended in the original protocol [3] to obtain a clearer background; this would however, lose the advantage of the accelerated staining procedure. Therefore, we aimed to establish a fast staining protocol that would address the gel background issue and retain the advantages of a fast procedure based on Dong’s protocol. We hypothesized that imidazole-zinc based reverse staining could play a role as background protection during CBS based on Dong’s protocol. We further optimized and validated the procedure using 1D and 2D SDS-PAGE. In this work, we present an optimized fast staining protocol with improved sensitivity, which requires just 60 to 75 minutes to complete protein visualization.

## Methods

### Materials

Coomassie Blue G, Acrylamide, and Bis-acrylamide were purchased from SERVA (Heidelberg, Germany); all other chemicals were purchased from Sigma-Aldrich (Prague, Czech Republic) if not otherwise specified.

### Samples

For 1D SDS-PAGE, samples of different BSA dilutions were prepared. BSA was dissolved in a sample buffer (50 mM Tris-HCl, 4% SDS, 100 mM DTT, 8.5% glycerol, and 0.01% bromophenol blue) to a 100 µg/ml concentration; the solution was further two-fold serially diluted. The final BSA samples loaded (10 µl) onto the gels corresponded to 1000, 500, 250, 125, 62.5, 31.25, 15.6, and 7.8 ng per lane, respectively. Bacterial whole cell lysate (*E. coli*) was purchased from Abcam (Cambridge, UK; ab5395). Peripheral blood mononuclear cells were isolated from whole blood using Histopaque-1077 (Sigma-Aldrich) according to manufacturer instructions. Cytosolic, nuclear, membrane, and cytoskeleton protein fractions were isolated using ProteoExtract Subcellular Proteome Extraction Kit (Merck, Darmstadt, Germany) according to manufacturer instructions.

For 2D SDS-PAGE, plasma and platelet samples were prepared. Blood was collected into tubes coated with EDTA, and plasma was obtained by centrifugation. Platelets were isolated according to the protocol by Reicheltová *et al*. [5]. Plasma was diluted 1:2 with PBS, platelets were resuspended in PBS (100 µl of PBS per a platelet pellet from 1 ml of whole blood), and cold acetone was added to both samples at a ratio of 1:4. Samples were then incubated at -20 °C overnight, centrifuged (15000 x g, 10 min, 4 °C), protein pellets rinsed with cold acetone; and the pellets were dissolved in a sample buffer for IEF (7 M urea, 2 M thiourea, 4% CHAPS, 65 mM DTT, 0.5% ampholytes, and a trace of bromophenol blue). Rat brain tissue lysate was purchased from Abnova (Taipei, Taiwan; L041W2) and diluted with the sample buffer for IEF; 50 ug of the sample per strip was used.

### Ethics Statement

The blood donor agreed to participate in this study and gave written informed consent. The study was approved by the Ethical Committee of the Institute of Hematology and Blood Transfusion in Prague. All samples were obtained and analyzed in accordance with the Ethical Committee regulations.

### Gel electrophoresis

IEF and SDS-PAGE were performed as described previously in detail [6], [7]. Briefly, 10 µl of each BSA sample per lane or 155 µl of sample per IPG strip (pI 4-7, 7 cm; Invitrogen, Carlsbad, CA, USA) were used for 1D or 2D SDS-PAGE, respectively. 3.75% stacking and 10% resolving gels were used at constant current of 25 mA per gel.

### Protein staining

Following electrophoresis, the gels were reverse stained with imidazole-zinc based on the protocol by Fernandez-Patron *et al*. [8]. Immediately after electrophoresis, the gels were incubated in a solution containing 200 mM imidazole and 0.1% SDS, then washed with water for 15 sec, and developed by incubation in 200 mM zinc sulfate for approximately 30 sec. Developing was stopped by a quick wash in excess of water. Reverse stained gels were then stained with CBS based on the protocol by Dong *et al*. [3]. Gels were boiled for 2 min in the staining solution (0.05% Coomassie Blue G in water) and then destained. The washing solution was replaced when it turned light blue – that was approximately every two to five minutes.

### Image analysis

Gels were scanned (16-bit grayscale), and the digitized images were processed with ImageJ [9] software (1D electropherograms). Gels from the same batch were compared for the influence of the temperature and concentration of the disodium EDTA washing solution on gel destaining. Three technical replicates were used for the estimation of sensitivity of different protocols using 1D SDS-PAGE. For 2D SDS-PAGE, Progenesis SameSpots software v3.2 (Nonlinear Dynamics, Newcastle upon Tyne, UK) was used to evaluate the number of protein spots (results are expressed as means ± standard deviations); three technical replicates were used for the analyses.

## Results and Discussion

Recently, it has been shown that a high gel background is the limiting factor of fast CBS [4]. In an attempt to resolve this issue, we hypothesized that imidazole-zinc based reverse staining could play the role of background protection during fast CBS. In reverse stained gels, the background is covered with the precipitate while protein bands or spots are not; therefore, access of the Coomassie Blue dye to the gel background during fast CBS might be limited. To test this assumption, gels were reverse stained with imidazole-zinc, followed by fast CBS, and then destained with an EDTA (disodium salt) solution at a boiling temperature (EDTA was chosen as a chelating agent removing imidazole-zinc precipitate, to maintain acidic conditions, and as being odorless at boiling temperatures). The background of gels was completely destained (to a clear transparent background) in 50 to 60 minutes. To maintain comparable conditions for the following protocol evaluation, the 60 minute washing time was chosen as no more destaining was observed following this interval. To test the sensitivity of the protocol, serially diluted BSA samples were used for 1D SDS-PAGE (from 1000 to 7.8 ng per lane), and the gels were treated as follows: (A) gels were stained according to Dong *et al*. [3] and destained six times for 1 min in boiling water, according to the original protocol; (B) gels were stained according to Dong *et al*. and destained for 1 hr in boiling water; (C) gels were stained according to Dong *et al*. and destained for 1 hr in a boiling EDTA solution; and (D) gels were stained according to our new proposed protocol – i.e., with imidazole-zinc reverse staining followed by fast CBS – and destained for 1 hr in a boiling EDTA solution.

**Figure 1:**
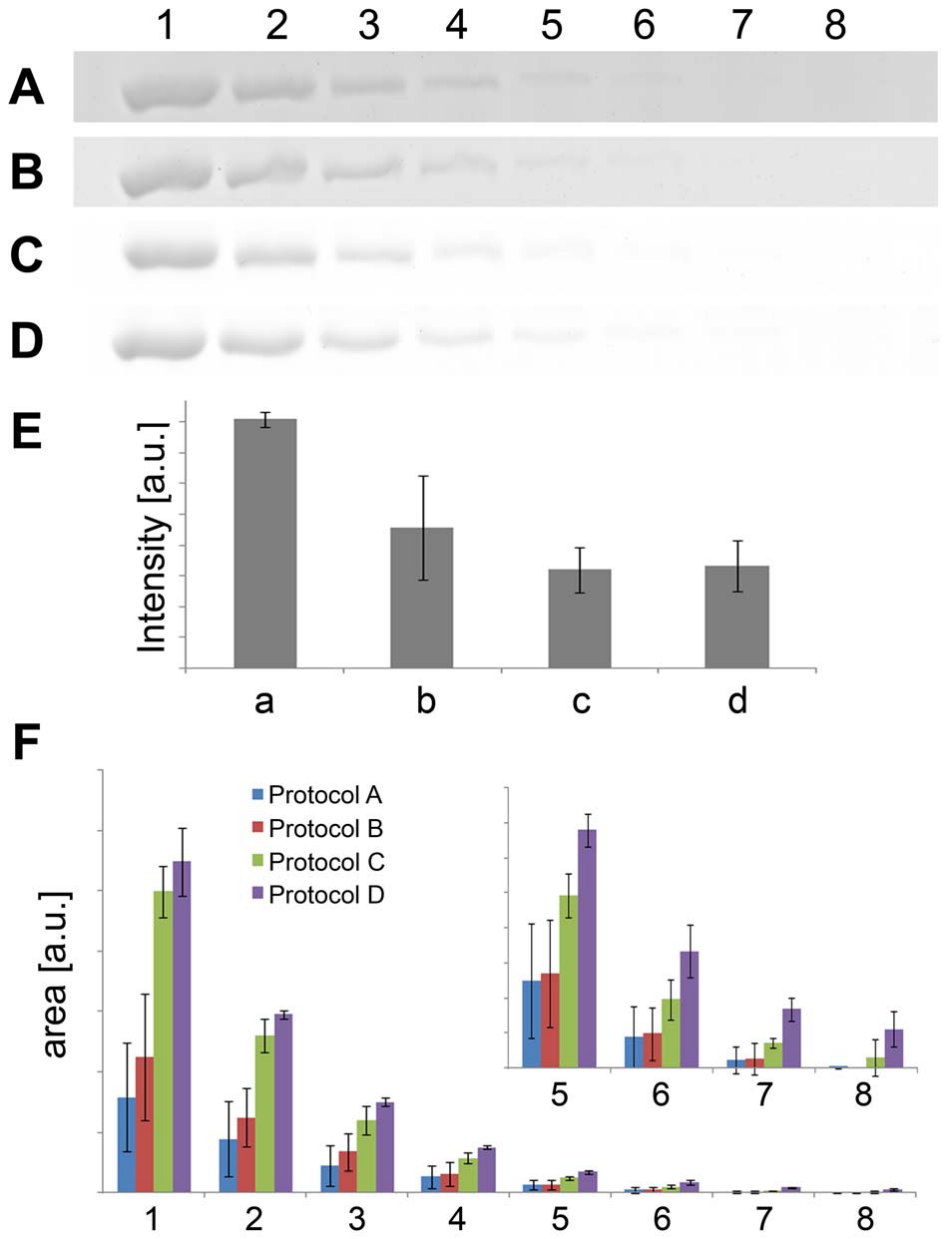
Comparing the effects of different protocols on the destaining of the gel background and detection of BSA bands. The effects of different protocols on gel background destaining was compared: gels were stained according to Dong *et al*. and destained six times for 1 min in boiling water, according to the original protocol (A); gels were stained according to Dong *et al*. and destained for 1 hr in boiling water (B); gels were stained according to Dong *et al*. and destained for 1 hr in a boiling EDTA solution (C); and gels were stained according to our new proposed protocol with imidazole-zinc reverse staining followed by fast CBS, and destained for 1 hr in a boiling EDTA solution (D). Gel background intensities were compared for all tested protocols (E), the results are expressed as mean values ± standard deviations from three independent experiments ; a, b, c, and d correspond to the above mentioned protocols A, B, C, and D, respectively. To estimate the differences in detection of BSA bands in gels stained according to the protocols A (blue), B (red), C (green), and D (purple), corresponding band areas were measured (F); the inset shows more detail illustration for lanes 5–8. The results are expressed as mean values ± standard deviations from three independent experiments. Two-fold serially diluted BSA samples were used for 1D SDS-PAGE and corresponded to 1000, 500, 250, 125, 62.5, 31.25, 15.6, and 7.8 ng per lanes 1–8, respectively.

The results are illustrated in Figure 1A-D; the dark gel background is obvious with gels treated according to the original Dong protocol (A), prolonging the washing step with boiling water to 1 hr reduced the gel background (B). Using a combination of imidazole-zinc and fast CB stainings (D), significant improvement could be observed, and a clear gel background was obtained. However, a clear gel background was also observed when the boiling EDTA solution (C) was used instead of water (B); moreover, nearly clear gel background can be achieved with water (B) when increasing the frequency of washing solution changes. Comparison of gel background intensity values achieved with the tested protocols (using three replicates) is shown in Figure 1E. It is apparent that prolonging the washing resulted in reduction of the gel background, particularly for protocols C and D utilizing the EDTA washing solution. In order to compare detection of BSA bands in gels stained according to appropriate protocols, corresponding band areas from three independent replicates were measured (Figure 1F). It is obvious that using the EDTA washing solution (protocols C and D) improved the detection when compared to both protocols A and B. Moreover, our new proposed protocol D was more effective in detection of bands, especially those containing low amounts of BSA. Thus, using an EDTA solution instead of water at a boiling temperature, and prolonging the washing to 50–60 min, considerably improved the sensitivity of the Dong protocol [3]; however, the combination of imidazole-zinc reverse and CB stainings followed by EDTA destaining further improved detection sensitivity.

**Figure 2:**
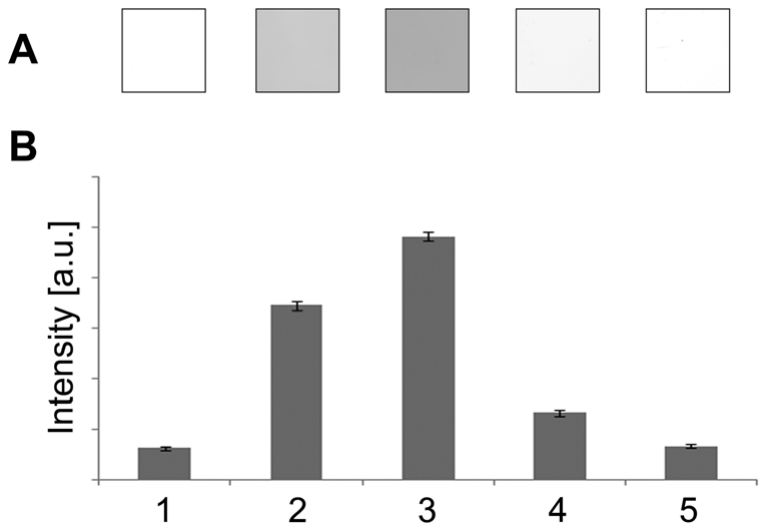
The influence of temperature on gel destaining. Gels were destained with a boiling EDTA destaining solution for 1 hr (1); the combination of six washes in a boiling EDTA solution for 1 min followed by a 1 hr room temperature EDTA washing solution (2); a room temperature EDTA washing solution for 1 hr (3) or overnight (4); and the combination of six washes in a boiling EDTA solution for 1 min followed by a 6 hr room temperature washing (5). Illustrations of gel backgrounds after destaining with appropriate procedures are shown (A). Average area intensity values and their standard deviations for gels destained using appropriate procedures are presented as estimated using ImageJ software (B).

The influence of the temperature of the EDTA washing solution on gel destaining was estimated (Figure 2). Gels were destained with: (i) a boiling solution, (ii) the combination of six washes in a boiling solution for 1 min followed by a room temperature washing solution, or (iii) a room temperature washing solution. While using the boiling EDTA solution (i) produces a transparent gel background in 50 to 60 min, washing with room temperature EDTA (iii) does not result in a clear background even after overnight washing. With the gels that had been washed six times for 1 min with a boiling EDTA solution and then followed by a room temperature solution washing (ii), a transparent gel background was achieved after six hours. Therefore, using the EDTA solution at a boiling temperature is of critical importance and significantly reduces the time required for the destaining step.

**Figure 3:**
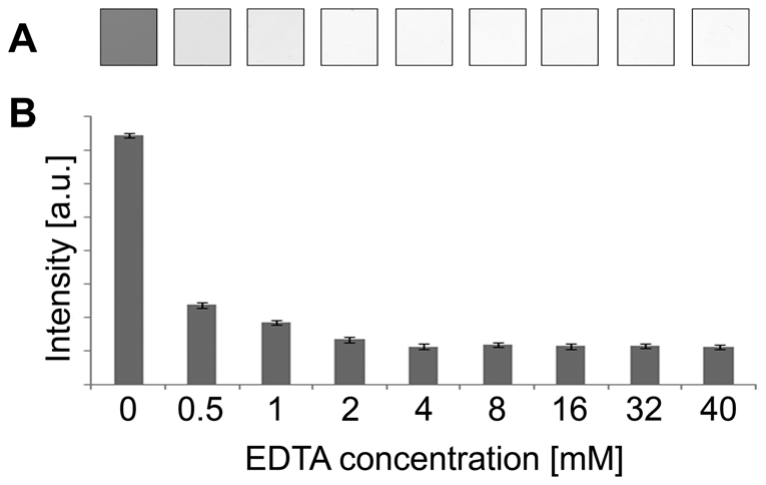
The influence of EDTA concentration on gel destaining. Illustrations of gel backgrounds after 1 hr incubation using different EDTA concentrations (0, 0.5, 1, 2, 4, 8, 16, 32, and 40 mM EDTA solutions, respectively) are shown (A). Average area intensity values and their standard deviations for gels destained with corresponding EDTA concentrations are presented as estimated using ImageJ software (B).

The influence of EDTA concentration on gel destaining was estimated; 0.5, 1, 2, 4, 8, 16, 32, and 40 mM EDTA solutions were used (Figure 3). After 1 hr incubation destaining was apparent using 1 mM EDTA; however, complete destaining was achieved using 4 mM EDTA. Higher EDTA concentrations seemed to slightly reduce destaining time (maximal decrease of 5 min during 1 hr incubation); however, using concentrations of EDTA more than 8 mM seemed to have no significant effect. Thus, using 2 mM EDTA provided clear gel backgrounds, but the time required exceeded 1 hr; increasing the EDTA concentration to 8 mM or higher reduced the washing time by a maximum of only 5 min. Therefore, keeping the protocol effective and at the same time low-cost, a 4 mM EDTA solution was consequently used as a destaining solution.

The final protocol is summarized as follows: immediately after electrophoresis, gels are incubated in a 200 mM imidazole/0.1% SDS solution for 10 min, then briefly rinsed with water for 15 sec, and developed by incubation in 200 mM of zinc sulfate for approximately 30 sec. Developing is stopped by rinsing in an excess of water. At this point the procedure can be interrupted, and the gels stored in water in a refrigerator for later use. In the next step, gels are boiled for 2 min in the staining solution (0.05% Coomassie Blue G in water) and destained with a 4 mM EDTA (disodium salt) solution at a boiling temperature until a transparent gel background is achieved (approximately 50 to 60 min); it is of critical importance to keep the washing solution at or just below the boiling point. The washing solution should be changed regularly as it turns light blue (approximately 3 to 5 min) – longer durations between changing of the solution may prolong the destaining process. The duration of gel destaining should not vary too much among batches as different gel background levels may be produced and thus hamper gel comparisons.

**Figure 4:**
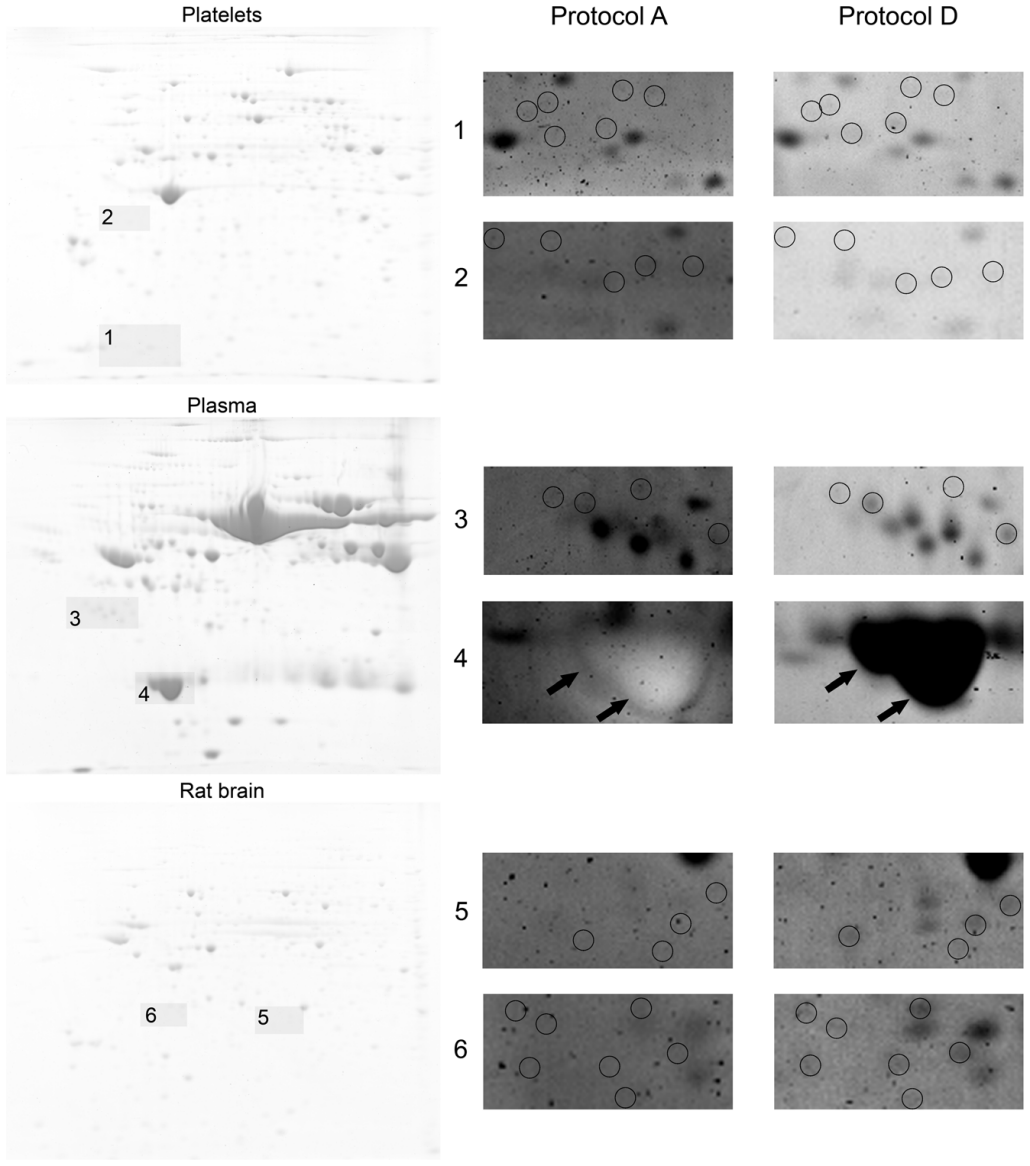
2D SDS-PAGE of blood platelet, undepleted plasma, and rat brain tissue samples. The effect of an appropriate protocol on gel background destaining and spot detection is illustrated using blood platelet, undepleted plasma, and rat brain tissue samples analyzed by 2D SDS-PAGE. The gels were processed according to the original Dong (Protocol A) and the final (Protocol D) protocols. Magnified insets were exported from the Progenesis SameSpots software (brightness and contrast of the insets were adjusted by the software) and correspond to the gray areas highlighted in the gels. Spots that were detected in gels stained according to the final protocol D only are indicated with circles or arrows.

**Figure 5:**
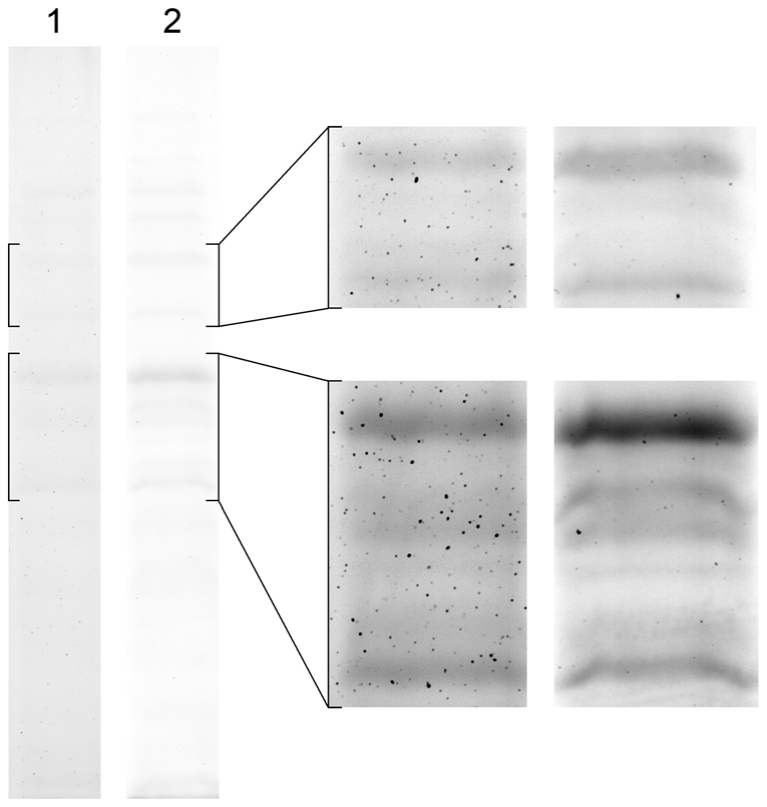
A comparison of bacterial (*E. coli*) proteins staining. Whole cell lysate (*E. coli*) was separated by 1D SDS-PAGE and gels were stained according to the original Dong protocol (lane 1) and according to our new proposed protocol with imidazole-zinc reverse staining followed by fast CBS and EDTA destaining (lane 2) to illustrate the effect of both protocols. Brightness and contrast of the magnified insets were adjusted for better illustrations.

**Figure 6:**
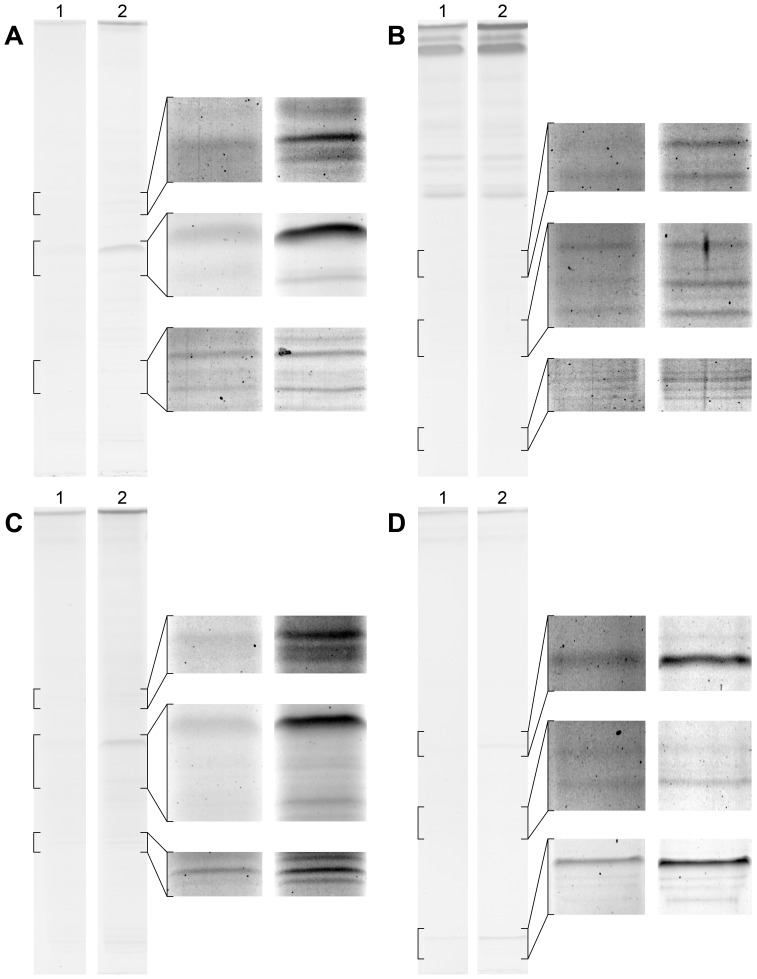
Staining of different protein fractions. 1D SDS-PAGE gels were stained according to the original Dong protocol (lanes 1) and according to our new proposed protocol with imidazole-zinc reverse staining followed by fast CBS and EDTA destaining (lanes 2) to illustrate the effect of both protocols. To compare various protein types four different peripheral blood mononuclear cell protein fractions were used: cytosolic (A), nuclear (B), membrane (C), and cytoskeleton (D) protein fractions. Brightness and contrast of the magnified insets were adjusted for better illustrations.

Using a 1D SDS-PAGE based approach together with BSA dilution is however, not predictive enough and is not satisfactory for 2D SDS-PAGE, capable of resolving hundreds to thousands of proteins [4]. Therefore, 2D SDS-PAGE of blood platelets, undepleted plasma samples, and rat brain tissue samples (Figure 4) was used to compare the number of protein spots; the original Dong protocol [3] and the final protocol combining imidazole-zinc and CB stainings were compared. 520±40, 886±83, and 497±40 spots were detected using the Dong protocol in the plasma, platelet, and rat brain tissue samples, respectively. Using the final protocol combining imidazole-zinc and CB stainings, 667±19, 1195±48, and 785±16 spots were detected in the plasma, platelet, and rat brain tissue samples, respectively. Therefore, there were 28%, 35%, and 58% increases in the number of detected spots in the plasma, platelet, and rat brain tissue samples, when the final protocol combining imidazole-zinc and CB stainings was used. The increase in the plasma sample corresponds to the previously reported difference between the number of detected spots when Dong’s protocol and colloidal CBS were compared [4]; a higher gel background was found to be the reason for the lower number of detected spots. Thus, these results suggest that using the combined strategy directly solves the problem of a higher background; and it is obviously suitable for 2D SDS-PAGE analyses, as it considerably increases the number of detectable spots. The results also point out that there may be another factor contributing to the difference in the number of detected spots. Several spots were found to disappear from the 2D spot pattern when the original Dong protocol was used, nevertheless, those spots were stained properly when our final protocol was used; an example is illustrated in Figure 4 (panel 4). It is likely that the missing spots/proteins were washed out from the gel during the staining and destaining steps. The final protocol prevented this phenomenon probably due to the previous imidazole-zinc staining of the gels that eliminated protein elution. In addition to 2D SDS-PAGE analyses, a comparison of bacterial (*E. coli*) and four different peripheral blood mononuclear cells protein fractions (cytosolic, nuclear, membrane, and cytoskeleton) was performed to further estimate the suitability of our new proposed protocol for different protein types. The new protocol seemed to be more effective in protein detection regardless of protein types; the results are illustrated in Figure 5 and Figure 6, respectively.

The combination of imidazole-zinc reverse and Coomassie dye-based stainings may be used in many cases. Imidazole-zinc staining can be used after gels have been treated with CBS to detect those spots or bands that were not detected using CBS alone [10]. Another approach may use imidazole-zinc staining first to detect as many spots as possible, followed by complete destaining of the gel and then using CBS; such an approach would enable maximal spot detection and quantification using Coomassie dye. However, this is the first time imidazole-zinc reverse staining is being used in combination with fast CBS; this protocol solves the problem of the high gel background after fast CBS, and thus increases the sensitivity of fast staining protocols based on Coomassie Blue dye. Moreover, the above described procedure can be discontinued after imidazole-zinc reverse staining; gels can be scanned and images of both stainings of the gel can be obtained. Reverse stained gels can also be stored in distilled water in a refrigerator, and a batch of gels can be further processed efficiently by treating them all with boiling CB at once.
